# Social and psychological factors of the suicidal tendencies of Chinese medical students

**DOI:** 10.1186/1751-0759-8-23

**Published:** 2014-10-18

**Authors:** Aiming Zheng, Zhilin Wang

**Affiliations:** 1 Mental Health Education and Research Center of Nanjing Medical University, No.140 Hanzhong Road, Nanjing 210009, P.R. China

**Keywords:** Chinese medical students, Suicidal tendency, Social factors, Psychological factors

## Abstract

**Background:**

Over the past few decades, concern about suicide by college students has been on the rise worldwide, in general and in China particularly. The main objective of this study is to investigate the effects of social and psychological factors on the suicidal tendencies of Chinese medical students.

**Findings:**

Of the 540 students surveyed, 48 had a suicidal tendency to some extent. The highest rate of suicide was observed for fourth-year students, followed by the fifth-year, first-year, third-year, and second-year students. Female students and students not satisfied with their major had a higher rate of suicidal tendency. However, mature coping strategies had a protective effect on suicide. The stepwise regression analysis shows that academic burden, grade, and introversion/extraversion are the most significant risk factors for the suicidal tendency of Chinese medical students.

**Conclusion:**

Suicide is affected by demographic risk factors as well as psychological factors. Our results lend support to a multi-factorial approach to the understanding and prevention of suicide by college students.

## Findings

Over the past few decades, concern about suicide by college students has been on the rise worldwide, in general and in China particularly. It was reported to be second only to accidental death as a cause of mortality in young men across the world [1] and the leading cause of death for those aged 15 to 34 years [2]. However, despite the considerable attention focused on this problem, much remains to be understood about the potential risk factors for suicide by college students.

It has been well established that suicide is a complex phenomenon associated with a host of biological, social, and psychological factors [3-5]. Another set of studies focus on the psychological and situational causes of suicidal behaviors. It is known that a wide variety of stressful events may serve as a trigger for suicide [6]. For instance, interpersonal problems with family members or other intimates are a typical stressor experienced by those who made serious suicide attempts [3,7]. A family history of suicide has been implicated as a significant risk factor for suicide independent of severe mental disorders, and the rate of suicide was twice as high in the families of suicide victims as in comparison families [8]. Other factors that are closely related to suicide include the psychological state and socioeconomic factors such as poverty, discrimination, etc.

In a narrow sense, suicide is the self-inflicted death of an individual with explicit or inferred intent to die [9]. It is thus reasonable to consider the completed suicide as the outcome of a complex process that begins with the idea of suicide, followed by active attempts and eventually the death of the individual. However, the majority of previous studies focused on the causes of the completed suicide, and less study has been done on the suicidal ideation and attempt. In this study, we examine the effects of demographic variables, personality, stressful life events, social support, and coping strategies on the suicidal tendency of medical students in China. It might be helpful for identifying individuals at high risk of suicidal behaviors.

A total of 600 first- to fifth-year medical students in three medical universities in mainland China were surveyed in September 2013 (the period of medical education in China is normally five years, including one- or two-year internship). At last, 566 questionnaires were collected and 540 responses were valid, which represented a valid response rate of 90%. The final sample consisted of 132 (24%) first-year students, 108 (20%) second-year students, 104 (19%) third-year students, 104 (19%) fourth-year students, and 92 (17%) fifth-year students. Of the 540 participants, 284 (53%) were male and 256 (47%) were female, with a mean age of 20.80 years (range, 15-25 years; *SD*, 1.36 years).

Beck Hopelessness Scale, Cattell’s Sixteen Personality Factor questionnaire (16PF) Questionnaire, Adolescent Self-rating Life Events checklist, social support rating scale and coping style questionnaire were used to evaluate the suicidal tendency, personality, stressful life events, social support and coping style [10-14].

All statistical analyses were performed using SPSS, version 13.0. The difference between the participants with or without suicidal tendency was compared using the chi-square or t-test. Stepwise regression was used to identify the factors contributing to the suicidal tendencies of the participants. A *p*-value less than 0.05 was considered statistically significant.

Of the 540 students, 48 (8.89%), students consisting of 12 males and 38 females (2.22% vs. 6.67%, *χ*^*2*^ = 8.09, *P* < 0.05), have had a suicidal tendency to some extent, including 12 first-year students (9.09%), 6 (5.56%) second-year students, 8 (7.69%) third-year students, 12 (11.54%) fourth-year students, and 10 (10.87%) fifth-year students. Table 1 shows that students satisfied with their major have a significantly lower rate of suicidal tendency than those not satisfied or neutral (*χ*^*2*^ = 27.65, *P* < 0.5). Table 2 shows that students with a suicidal tendency have more interpersonal and academic problems (*P* < 0.05). Table 3 shows that students with a suicidal tendency have a lower score for fantasy and avoidance and a higher score for the self-accusation (*P* < 0.05). Table 4 shows that students with a suicidal tendency have a lower score for warmth, dominance, liveliness, social boldness, introversion/extraversion and a higher score for vigilance (*P* < 0.05). However, there is no significant difference in problem solving, appealing for help, rationalization, other aspects of stressful life events, economic background, and social support (*P* > 0.05).

**Table 1:** Major satisfaction and suicidal tendencies

**Major satisfaction**	**With suicidal tendencies (*n*** **= 48)**	**Without suicidal tendencies (*n*** **= 492)**	***χ***^***2***^	***p***
Does not matter	6 (12.50)	204 (41.46)	27.65	0.0018
Satisfied	10 (20.83)	194 (39.43)		
Dissatisfied	32 (66.67)	94 (19.11)		

**Table 2:** **Stressful life events and suicidal tendencies **$\left(\bar{x}\pm s\right)$

**Stressful life events**	**With suicidal tendencies (*n*** **= 48)**	**Without suicidal tendencies (*n*** **= 492)**	***t***	***p***
Interpersonal	13.25 ± 0.55	9.85 ± 1.48	11.287	<0.001
Academic	1.48 ± 0.76	0.56 ± 0.70	2.552	0.008
Punishment	0.49 ± 0.43	0.53 ± 0.54	-0.303	0.762
Loss of intimate or property	1.18 ± 1.04	1.18 ± 1.10	0.008	0.993
Physical disease	0.63 ± 0.52	0.83 ± 0.55	-1.780	0.076
Others	0.67 ± 0.61	0.65 ± 0.64	0.157	0.875

**Table 3:** **Coping styles and suicidal tendencies **$\left(\bar{x}\pm s\right)$

**Coping style**	**With suicidal tendencies (*n*** **= 48)**	**Without suicidal tendencies (*n*** **= 492)**	***t***	***p***
Problem solving	0.77 ± 0.18	0.81 ± 0.15	1.071	0.285
Self-accusation	0.29 ± 0.12	0.19 ± 0.26	2.552	0.012
Appealing for help	0.52 ± 0.27	0.59 ± 0.26	-1.281	0.201
Fantasy	0.36 ± 0.23	0.49 ± 0.21	-2.883	0.004
Avoidance	0.31 ± 0.17	0.42 ± 0.19	-2.751	0.006
Rationalization	0.38 ± 0.12	0.39 ± 0.18	-0.325	0.745

**Table 4:** **16-PF and suicidal tendencies **$\left(\bar{x}\pm s\right)$

**16-PF**	**With suicidal tendencies (*n*** **= 48)**	**Without suicidal tendencies (*n*** **= 492)**	***t***	***p***
Warmth (A)	3.00 ± 1.18	4.51 ± 1.95	5.271	<0.001
Reasoning (B)	6.64 ± 1.63	6.59 ± 1.53	0.211	0.830
Emotional stability (C)	6.09 ± 2.12	6.21 ± 2.10	0.382	0.706
Dominance (E)	3.00 ± 1.61	3.96 ± 1.60	3.973	<0.001
Liveliness (F)	4.55 ± 1.63	5.33 ± 1.75	2.964	0.003
Rule-consciousness (G)	5.82 ± 1.89	5.88 ± 1.95	0.198	0.838
Social boldness (H)	4.18 ± 1.54	5.22 ± 1.59	5.341	<0.001
Sensitivity (I)	5.73 ± 1.68	5.76 ± 1.59	0.118	0.901
Vigilance (L)	5.18 ± 1.65	4.27 ± 2.41	2.561	0.011
Abstractedness (M)	6.09 ± 1.81	6.11 ± 1.78	0.072	0.941
Privateness (N),	5.18 ± 1.67	4.61 ± 1.97	1.938	0.053
Apprehension (O)	6.18 ± 2.36	5.91 ± 2.06	0.862	0.393
Openness to change (Q1)	5.64 ± 2.58	5.27 ± 1.91	1.242	0.217
Self-reliance (Q2)	5.91 ± 1.81	5.35 ± 1.95	1.911	0.057
Perfection (Q3)	6.00 ± 1.26	5.82 ± 1.47	0.818	0.413
Tension (Q4)	5.64 ± 1.63	5.43 ± 2.03	0.690	0.487
Adaptation/anxiety	5.53 ± 2.23	5.41 ± 1.91	0.412	0.683
Introversion/extraversion	3.05 ± 1.74	4.70 ± 1.84	5.959	<0.001
Affection/alertness	5.57 ± 1.48	5.23 ± 1.59	1.422	0.156
Cowardice/determination	5.58 ± 1.65	5.14 ± 1.70	1.721	0.088
Mental health	20.64 ± 6.04	21.99 ± 6.32	1.421	0.157
Professional achievement	55.55 ± 8.19	54.96 ± 9.16	0.432	0.668
High creativity	90.55 ± 8.36	87.84 ± 11.18	1.632	0.102
Ability to adapt to unfamiliar conditions	25.09 ± 3.01	24.95 ± 3.77	0.254	0.803

The total scores for a suicidal tendency were regressed for sex, satisfaction with major, grade, economical background, stressful life events, social support, coping style, and 16PF scores. Table 5 shows that academic burden, grade, and introversion/extraversion enter the regression model and account for a significant proportion of the variance in suicidal tendency (*β* = -0.34, -0.22, -0.22, *P* < 0.05, respectively).

**Table 5:** Multiple regression analysis results

	**B**	**SE B**	***β***	**R2**	**△R2**	***t***	***p***
				**0.216**	**0.189**		
Academic burden	-1.005	0.276	-0.344			-3.641	<0.001
Grade	-0.472	0.204	-0.219			-2.314	0.023
Introversion/extraversion	-0.207	0.090	-0.217			-2.301	0.024

This study highlights the necessity to take into account some demographic risk factors, such as grade, sex, and major satisfaction. The highest rate of suicide was observed in the fourth-year students, followed by the fifth-year, first-year, third-year, and second-year students. The fourth-year students are in a position of seriously considering their future career as a medical practitioner, and most of them have been fully engaged in the internship. While facing the substantial changes of lifestyle and uncertainty about the future, they may be drawn to the idea of committing suicide as a way to achieve emotional relief or escaping stress escaping. On the other sidehand, the first-year students who are commencing their college studies may have adaptation problems. The fifth-year students become vulnerable to suicide due to the frustration encountered in job-hunting. These students are at high risk of suicide and thus in need of attention. Second, female students appear to be more likely than males to develop a suicide tendency due to the. Female Chinese students tend to be sensitive, shy, fanciful, dependent, and reluctant to express their emotions (especially negative emotions) outwardly because of their cultural background. Third, students not satisfied with their major have a higher rate of suicidal tendency. The results clearly point to a need for reform of the admission policy currently practiced in China. A better alternative is to let students choose their major one or two years after entrance to college instead of before the National Higher Education Entrance Examination, which might reduce suicides due to the mismatch between preference and reality.

Stressful life events have been identified as a precursor to suicidal behavior [15-17]. The results show that individuals with a suicidal tendency have more interpersonal and academic problems. Previous studies [18-20] have also indicated that suicide is related to personality disorder, increased negative life events, lack of social support, and immature coping strategies.

The findings of present study should be interpreted in the context of its limitations. First, the present study is limited to some extent by the exclusive use of Chinese participants, thus the generalizability of the findings would be significantly enhanced by replication with participants with different backgrounds. Second, suicide is a complex phenomenon affected by a variety of biological, social, and psychological factors, while some other risk factors, such as social transformation, educational evaluation system and multiple comparison of each question, were not considered in this study.

We conclude that demographic risk factors such as grade, sex, and satisfaction with major may be related to suicide. Specifically, fourth-year female students and those not satisfied with their major have a higher rate of suicidal tendency. In addition, students high in stressful life events and adopting maladaptive coping strategies are more likely to commit suicide. Our results lend strong support to a multi-factorial approach to the understanding and prevention of suicide by college students, and the best way to cope with it is to address the major risk factors in an integrated manner.

## Ethical approval

Participants for this study were all adult volunteer students. They signed a formal agreement before enrolled in the research. All students were advised that they could withdraw, or not participate, without any effect on their academic progress. The information collected was strictly preserved, and whole procedure was approved by the Ethical Committee of Nanjing Medical University, Jiangsu, China.

## Abbreviations

16PF: Sixteen personality factor questionnaire.

## Competing interests

The authors declare that they have no competing interest.

## Authors’ contributions

Conceived and designed the experiments: AZ. Performed the experiments: AZ, ZW. Analyzed the data and wrote the manuscript: AZ. Both authors read and approved the final manuscript.
